# Plasma membrane receptor-like kinase leaf panicle 2 acts downstream of the DROUGHT AND SALT TOLERANCE transcription factor to regulate drought sensitivity in rice

**DOI:** 10.1093/jxb/eru417

**Published:** 2014-11-10

**Authors:** Fuqing Wu, Peike Sheng, Junjie Tan, Xiuling Chen, Guangwen Lu, Weiwei Ma, Yueqin Heng, Qibing Lin, Shanshan Zhu, Jiulin Wang, Jie Wang, Xiuping Guo, Xin Zhang, Cailin Lei, Jianmin Wan

**Affiliations:** ^1^National Key Facility for Crop Gene Resources and Genetic Improvement, Institute of Crop Science, Chinese Academy of Agricultural Sciences, Beijing 100081, PR China; ^2^National Key Laboratory for Crop Genetics and Germplasm Enhancement, Nanjing Agricultural University, Nanjing 210095, PR China

**Keywords:** Abiotic stress, leaf panicle 2, LRR, *Oryza sativa*, RLK, water channel.

## Abstract

Drought is a recurring climatic hazard that reduces the crop yields. To avoid the negative effects of drought on crop production, extensive efforts have been devoted to investigating the complex mechanisms of gene expression and signal transduction during drought stress. Receptor-like kinases (RLKs) play important roles in perceiving extracellular stimuli and activating downstream signalling responses. The rice genome contains >1100 RLK genes, of which only two are reported to function in drought stress. A leucine-rich repeat (LRR)-RLK gene named *Leaf Panicle 2* (*LP2*) was previously found to be strongly expressed in leaves and other photosynthetic tissues, but its function remains unclear. In the present study, it was shown that the expression of *LP2* was down-regulated by drought and abscisic acid (ABA). Transgenic plants overexpressing *LP2* accumulated less H_2_O_2_, had more open stomata in leaves, and showed hypersensitivity to drought stress. Further investigation revealed that transcription of *LP2* was directly regulated by the zinc finger transcription factor DROUGHT AND SALT TOLERANCE (DST). In addition, LP2 was identified as a functional kinase localized to the plasma membrane and interacted with the drought-responsive aquaporin proteins OsPIP1; 1, OsPIP1; 3, and OsPIP2; 3. Thus, the findings provided evidence that the LRR-RLK LP2, transcriptionally regulated by the drought-related transcription factor DST, served as a negative regulator in drought response.

## Highlight

A leucine-rich repeat receptor-like kinase LP2 is transcriptionally controlled by zinc finger protein DST and serves as a negative regulator in drought response

## Introduction

Plants are sessile organisms and are frequently exposed to biotic and abiotic stresses during their life cycle. Among abiotic stresses, drought is one of the most common environmental factors that limit crop productivity, especially in Asia where at least 23 million ha of rice (20% of the total world rice area) are drought prone (Pandey *et al.*, 2000). The economic costs of drought can be enormous. For example, the 2004 drought in Thailand was estimated to have affected 2 million ha of crop area, and had effects on >8 million people (Pandey *et al.*, 2000). Drought affects the development and grain yield of rice at different growth stages, such as germination, vegetative growth, spikelet development and fertility, and grain filling (Wade *et al.*, 1999), by suppressing leaf expansion, tillering and mid-day photosynthesis, and by reducing photosynthetic rates and leaf area due to early senescence (Nooden and Leopold, 1988; Kramer and Boyer, 1995). Considerable effort has been made to discover the key genes and signalling pathways involved in plant responses to drought stress. Detailed analysis will help in understanding the mechanism of drought signalling transduction and lay a foundation for engineering strategies to improve stress tolerance of crops around the world.

Perception and processing of extracellular signals through plasma membrane receptor-like protein kinases (RLKs) in plants lead to alterations in the concentrations of cellular ions and molecules that activate protein phosphorylation pathways. This in turn regulates expression of stress-responsive genes to generate physiological, biochemical, and other adaptive responses that reduce or eliminate the problem (Lee *et al.*, 2011; Xing *et al.*, 2011). RLKs belong to the serine/threonine protein kinase family. Through phosphorylation and dephosphorylation of the Ser/Thr residues, RLKs convert external signals to intracellular cytoplasmic signals (Stone and Walker, 1995; Morillo and Tax, 2006). RLKs comprise a major gene family in plants, with at least 610 members in *Arabidopsis* and ~1132 members in rice (Shiu *et al.*, 2004; Morillo and Tax, 2006). Based on the N-terminal extracellular domains, RLKs fall into multiple subfamilies, and those containing a leucine-rich repeat domain (LRR-RLKs) constitute the largest subfamily in plants. In rice, ~309 LRR-RLK genes have been identified and classified into five subgroups based on phylogenetic analysis (Sun and Wang, 2011). LRR-RLKs are implicated in diverse signalling events that lead to such things as resistance to pathogens, plant development, and growth (Sun and Wang, 2011). Accumulating data suggest that LRR-RLKs also play an important role in regulating environmental stress responses. Five LRR-RLKs involved in stress response in plants have been identified at the molecular level. *Srlk* (*Salt-induced Receptor-Like Kinase*) encoding an LRR kinase in *Medicago truncatula* was reported to regulate the response of roots to salt stress (de Lorenzo *et al.*, 2009). *GHR1* (*GUARD CELL HYDROGEN PEROXIDE-RESISTANT1*) mediates abscisic acid (ABA) and hydrogen peroxide (H_2_O_2_)-regulated stomatal movement in *Arabidopsis* (Hua *et al.*, 2012). *Arabidopsis RLK7* is involved in the control of germination speed and tolerance to oxidation stress (Pitorre *et al.*, 2010). *RPK1* (*receptor-like protein kinase 1*) functions as a positive regulator of ABA signalling; transgenic *Arabidopsis* plants overexpressing *RPK1* showed increased ABA sensitivity in root growth and stomatal closure, and displayed less transpirational water loss (Osakabe *et al.*, 2010).

To date, only two LRR-RLK members in rice are reported to play a role in response to environmental stress. *OsSIK1* is expressed mainly in stems and spikelets, and its expression is induced by salt, drought, or H_2_O_2_ treatment. Transgenic rice plants overexpressing *OsSIK1* showed higher tolerance to salt and drought stresses, whereas knock-out mutants were sensitive to the same stresses (Ouyang *et al.*, 2010). *OsSIK1*-overexpressing plants accumulated much less H_2_O_2_, and stomatal density was reduced by 8.4–17.8% in leaves (Ouyang *et al.*, 2010). OsSIK2 is an S-domain RLK and is expressed mainly in rice leaves and leaf sheaths (Chen *et al.*, 2013). *OsSIK2* was induced by NaCl, drought, cold, dark, and ABA treatment, and transgenic plants overexpressing *OsSIK2* exhibited enhanced tolerance to salt and drought stress compared with controls (Chen *et al.*, 2013). Although the importance of RLK genes in abiotic stress has been well recognized in both monocots and dicots, the regulatory mechanisms governing their expression remain elusive.

Aquaporins, known as membrane intrinsic proteins (MIPs), are membrane channels that facilitate the transport of water and small neutral molecules (Maurel *et al.*, 2008; Li *et al.*, 2014). In plants, aquaporins regulate various physiological processes such as leaflet movement, CO_2_ assimilation, osmoregulation, uptake of mineral elements, and stomatal conductance (Gerbeau *et al.*, 1999; Hanba *et al.*, 2004; Siefritz *et al.*, 2004; Ma *et al.*, 2006). Aquaporins are also reported to be involved in various abiotic stresses, such as drought, salinity, and low temperature (Lian *et al.*, 2004; Prak *et al.*, 2008; Matsumoto *et al.*, 2009; Mosa *et al.*, 2012). There are 35 and 39 MIP members in the *Arabidopsis* and rice genomes, respectively (Wallace and Roberts, 2004; Sakurai *et al.*, 2005). On the basis of sequence homology and localization, plant MIPs are classified into four subfamilies: plasma membrane intrinsic proteins (PIPs), tonoplast intrinsic proteins (TIPs), small basic intrinsic proteins (SIPs), and nodulin-26 like intrinsic proteins (NIPs) (Li *et al.*, 2014). The PIP subfamily can be further divided into two phylogenetic subgroups PIP1 and PIP2. PIP1 isoforms generally have very low or no water channel activity, whereas PIP2 subgroup members possess high water channel activity (Kammerloher *et al.*, 1994; Weig *et al.*, 1997). The activity of PIPs is regulated by post-translational modifications and protein interactions (Chaumont and Tyerman, 2014). In a phosphoproteomic study on rice plasma membranes, several aquaporins expressed in shoots were phosphorylated (Whiteman *et al.*, 2008). However, knowledge of the protein kinases that phosphorylate aquaporins is still poorly understood.

In this study, we carried out a functional analysis on an LRR-RLK LP2 in rice. Expression of *LP2* was down-regulated by various abiotic stresses. Transgenic plants overexpressing *LP2* accumulated reduced levels of H_2_O_2_ and were sensitive to drought stress. Further analyses indicated that *LP2* was transcriptionally regulated by a previously reported C2H2 zinc finger transcriptional factor DST (DROUGHT AND SALT TOLERANCE). It was also shown that LP2 interacted *in vivo* with three PIPs. It is reported herein that DST-regulated LRR-RLK LP2 participates in drought stress response.

## Materials and methods

### Plant materials and growth conditions

Rice (*Oryza sativa*) japonica cv. Nipponbare was used in this study. Drought treatment was applied mainly according to published methods (Ning *et al.*, 2011) with some modifications. Seeds of wild-type and LP2-overexpressing transgenic plants were soaked in water for 2 d, and left for another 5 d. Seven-day-old seedlings were planted in small pots containing the same amount of soil and grown in a phytotron (PAR 384 μmol m^–2^ s^–1^, 25 ºC day/20 ºC darkness, 12h photoperiod). After 2 weeks, water was removed from the tray to begin drought treatment. Plant phenotypes were observed after 6, 7, and 8 d. Nine days after treatment, the plants were moved back to the watered trays for recovery. Survival rates of the transgenic lines and wild-type plants were recorded 4 d after rehydration. The drought stress experiment was performed at least three times.

### Generation of *LP2*- and *DST*-overexpressing transgenic rice plants

For construction of the *LP2* overexpression vector, the full-length cDNA of *LP2* was amplified and inserted into the pCUbi1390 vector under control of the *Zea mays ubiquitin* promoter. For construction of the *DST* overexpression vector, the full-length cDNA of *DST* was amplified and inserted into pCUbi1390 with a 3×FLAG-tag at the C-terminus. The constructs were introduced into Nipponbare by *Agrobacterium tumefaciens*-mediated transformation as described previously (Hiei *et al.*, 1994). Transgenic rice lines and their progeny were grown in paddy fields in Beijing (39°54’N, summer season, temperate climate) or Hainan (18°16’N, winter season, subtropical climate) under local growing conditions. Seeds of T_2_ homozygous overexpression lines were subjected to molecular and phenotypic analyses. The primer sequences used for the construction of this vector are listed in Supplementary Table S1 available at *JXB* online.

### Quantitative measurement of H_2_O_2_

H_2_O_2_ contents were determined with a Hydrogen Peroxide assay kit (Beyotime, S0038) according to the manufacturer’s protocol.

### Imaging of rice stomata and measurement of stomatal density

Imaging of rice stomata was conducted as described previously (Huang *et al.*, 2009) with some modifications. Leaves of 4-week-old plants were detached and immediately put between cardboard sheets to avoid curling, and frozen with liquid nitrogen. Stomatal images were obtained using an FEI Quanta 200 environmental scanning electron microscope (ESEM; FEI Corporation, The Netherlands).

### Subcellular localization of LP2–GFP proteins

The coding sequence of *LP2* was ampliﬁed and cloned into the N-terminus of green fluorescent protein (GFP) under control of the *Cauliflower mosaic virus* (CaMV) 35S promoter in the transient expression vector pA7-GFP, generating recombinant pA7-LP2-GFP. The recombinant vector was then transformed into rice protoplasts according to protocols described previously (Chen *et al.*, 2006), or into onion (*Allium* spp.) epidermal cells via shotgun bombardment (PDS-1000/He; Bio-Rad). The GFP signal was visualized using a confocal laser-scanning microscope (LSM 700; Carl Zeiss).

### RNA preparation and qPCR analysis

Total rice RNA was extracted with an RNA Prep Pure Kit (Zymo Research) according to the manufacturer’s instructions. First-strand cDNA was synthesized from 2 μg of total RNA using a SuperScript II Kit (TaKaRa). Primer pairs used for qPCR are listed in Supplementary Table S1 at *JXB* online. qPCR analysis was conducted using an ABI7500 fast qPCR system with the SYBR Premix Ex Taq (TaKaRa; RR041A). The procedure was as follows: initial polymerase activation for 30 s at 95 °C followed by 40 cycles of 95 °C for 5 s and 60 °C for 34 s. For each sample, qPCR was performed with three technical replicates on three biological replicates. The 2^–ΔΔCT^ method was used to analyse relative transcript levels in gene expression (Livak and Schmittgen, 2001). Primers used for ChIP-PCR are listed in Supplementary Table S1. For detecting the expression level of *LP2* in the *dst* mutant, 4-week-old plants were used for RNA isolation.

### Yeast one-hybrid assay

The assay was carried out as described previously (Gao *et al.*, 2013). To generate AD-DST, the full-length DST coding sequence was amplified by reverese transcription–PCR (RT–PCR) from the Nipponbare cultivar and ligated into the pB42AD vector (Clontech) digested with *Eco*RI. To generate LP2p::LacZ reporter genes, various fragments of the LP2 promoter were amplified from Nipponbare genomic DNA and inserted into the corresponding sites in the reporter plasmid pLacZi. Plasmids were co-transformed into yeast strain EGY48. Transformants were grown on SD/Trp-/Ura plates for 48h and then transferred onto X- gal (5-bromo-4-chloro-3-indolyl-β-d-galactopyranoside) plates for blue colour development.

### ChIP-PCR assay

Chromatin immunoprecipitation (ChIP) assays were performed as described previously (Zhang *et al.*, 2012) with some modifications. Approximately 2g of LP2-FLAG-overexpressing transgenic callus was ground to fine powder with liquid nitrogen for further analysis. Monoclonal mouse anti-Flag antibody (Sigma, St. Louis, MO, USA) was used in a ChIP assay. Chromatin precipitated with a pre-immune serum was used as the negative control, whereas the chromatin before precipitation was used as an input control. Primers used for ChIP-PCR are listed in Supplementary Table S1 at *JXB* online.

### Firefly luciferase complementation imaging assay

The assay was carried out as described previously (Hua *et al.*, 2012). LP2 and RAR1 sequences were fused upstream of N-Luc in the pCAMBIA-NLuc vector, and OsPIP1; 1, OsPIP1; 3, OsPIP2; 3, as well as AvrB were fused downstream of C-Luc in the pCAMBIACLuc vector.

## Results

### Identification and sequence analysis of the *LP2* gene

**Fig. 1.**
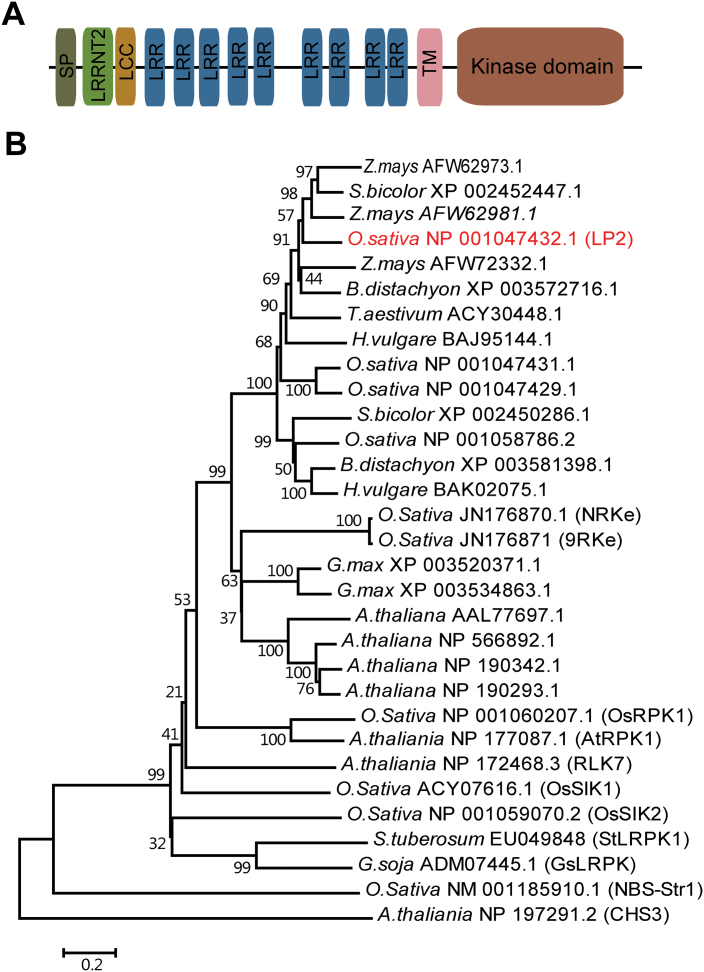
Protein domain structure and phylogenetic analysis of *LP2*. (A) Schematic depiction of the major domains in LP2. (B) Phylogenetic analysis of *LP2* and other LRR receptor-like kinases constructed by the Neighbor–Joining method. The LP2 protein is shown in red.

The expression level of an LRR-RLK was highly reduced in an analysis of microarray data from the drought-tolerant *dst* mutant (Huang *et al.*, 2009). The gene, named *Leaf Panicle 2* (*LP2*; Os02g40240), was strongly expressed in leaves and other photosynthetic tissues (Thilmony *et al.*, 2009). To characterize further the function of LP2 in drought response, the protein sequence was analysed. The protein contains a putative N-terminal signal peptide (SP), a putative extracellular domain containing 11 LRR domains (one LRRNT_2 domain, an LCC domain, and nine LRR domains), a transmembrane domain (TM), and an S_TKc intracellular kinase domain identified by the SMART program (Fig. 1A). BLAST searches of available genome sequences revealed that a homologue sharing the highest sequence homology with *LP2* (47.76% amino acid identity) was from *Brachypodium distachyon*. Close homologues of *LP2* were also identified in monocots such as *Zea mays*, *Sorghum bicolor*, *Triticum aestivum*, *Hordeum vulgare*, and *Oryza sativa* (Fig. 1B); however, the functions of these genes were largely unknown. There are several LRR-RLKs in *O. sativa*, *Arabidopsis thaliana*, and *Glycine soja* that function in abiotic stress (Wu *et al.*, 2009; Ouyang *et al.*, 2010; Pitorre *et al.*, 2010; Yang *et al.*, 2010; Zhang *et al.*, 2011; Ray *et al.*, 2012; Chen *et al.*, 2013; Shi *et al.*, 2014; Yang *et al.*, 2014). Among them, OsSIK1 and OsSIK2 were reported to improve drought and salt stress tolerance in rice plants (Ouyang *et al.*, 2010; Chen *et al.*, 2013), but OsSIK1 and OsSIK2 share only 30.56% and 18.04% amino acid identities with LP2, respectively.

### *LP2* is preferentially expressed in vegetative tissues and LP2 is a functional kinase localized to the plasma membrane

**Fig. 2.**
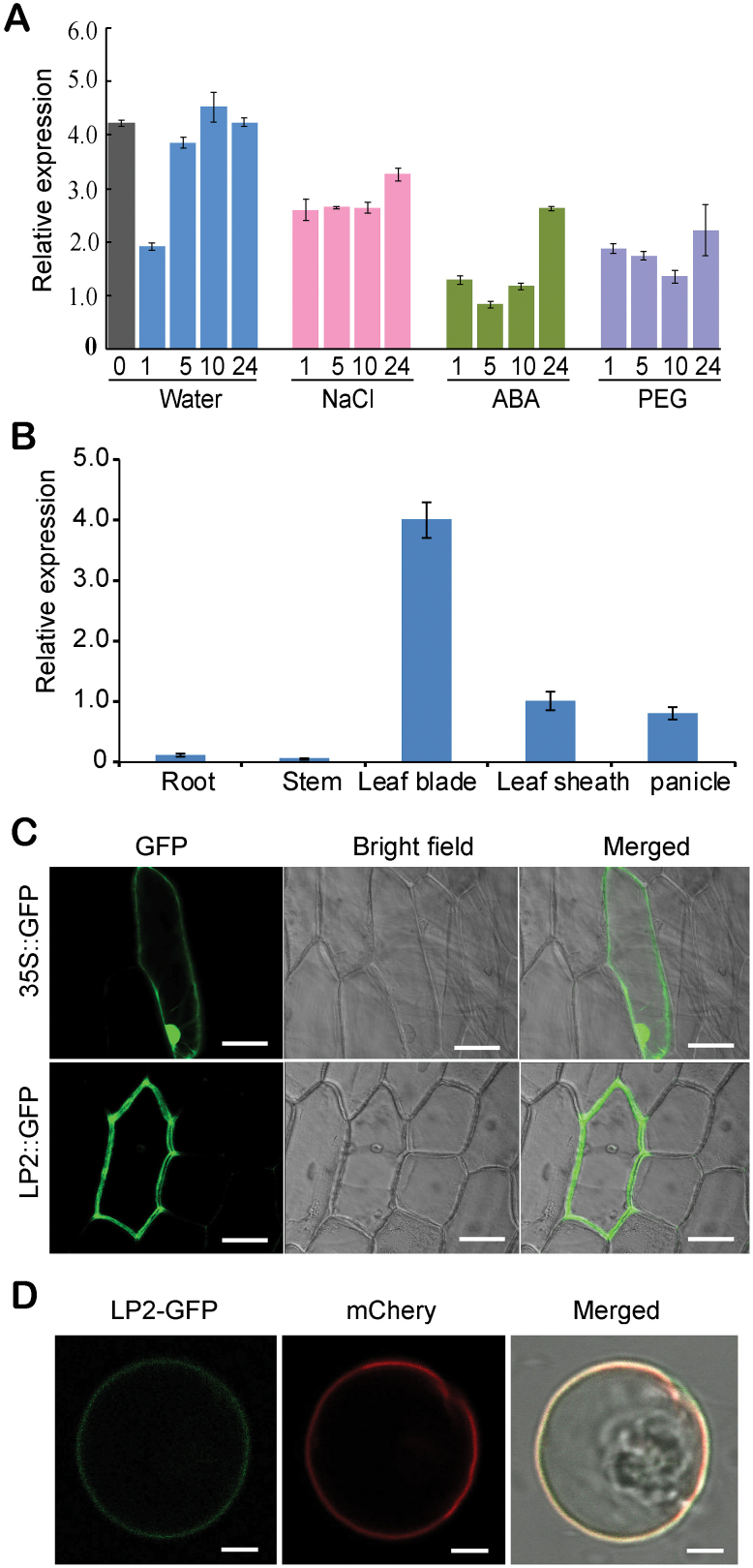
Transcript analysis and subcellular localization of LP2. (A) Determination of *LP2* transcript levels by qRT–PCR in response to water, NaCl, ABA, and PEG. (B) Tissue-specific expression pattern of *LP2* (means ±SD, *n*=3). (C) Subcellular localization of the fused LP2–GFP in onion epidermal cells. Bars=100 μm. (D) Subcellular localization of the fused LP2–GFP in rice protoplasts. Bar=5 μm.

To confirm whether the expression of *LP2* is regulated by abiotic stress, quantitative RT–PCR (qRT–PCR) was used to investigate the transcriptional profile of *LP2* under various stress conditions. *LP2* was down-regulated after 1h of water treatment, but then steadily increased to the original level after 5h (Fig. 2A). However, expression was highly inhibited by polyethylene glycol (PEG) and ABA treatments after 24h (Fig. 2A). A previous study showed that *LP2* was highly expressed in leaves and other photosynthetic tissues (Thilmony *et al.*, 2009). The tissue expression profiles of *LP2* were investigated by qRT–PCR analyses. *LP2* was preferentially expressed in leaf blades and sheaths, moderately expressed in young panicles, but was not detectable in other tissues, such as roots and stems (Fig. 2B). Transient expression of an LP2–GFP fusion protein in onion epidermal cells and rice protoplasts indicated that LP2–GFP is localized in the plasma membrane (Fig. 2C, D), suggesting a potential role for LP2 in the plasma membrane.

**Fig. 3.**
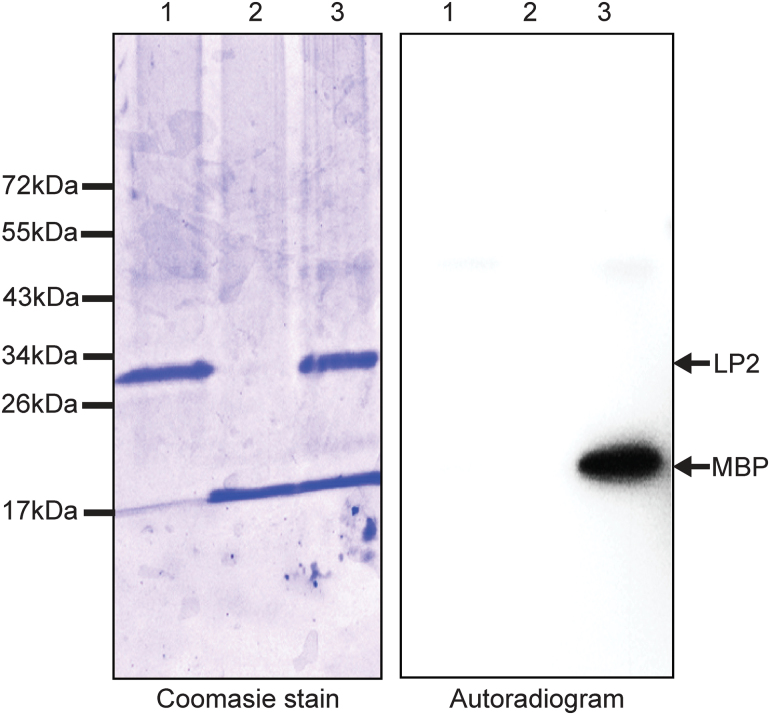
*In vitro* kinase assays with the LP2 kinase domain using MBP as substrate. Protein was visualized by Coomassie Blue staining (left panel), and protein phosphorylation was detected by autoradiography ([^32^P], right panel). The experiment was repeated twice with similar results.

To determine if LP2 has kinase activity, the intracellular kinase domain (residues 682–978) of LP2 fused to a 6× His tag was expressed in *Escherichia coli* strain *BL21*. An *in vitro* kinase assay was then performed with myelin basic protein (MBP) as substrate. The kinase domain showed no autophosphorylation activity, although it phosphorylated the artificial substrate MBP (Fig. 3).

### Up-regulation of *LP2* expression leads to drought sensitivity of transgenic plants

**Fig. 4.**
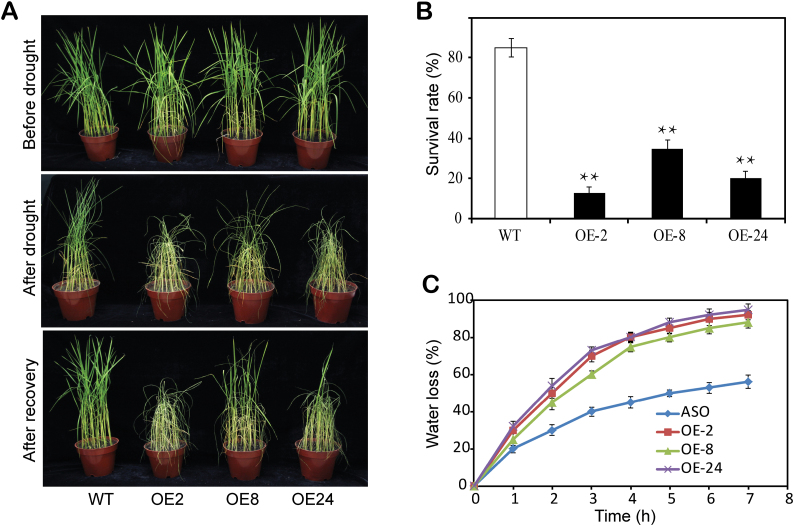
Drought tolerance testing of *LP2*-overexpressing rice. (A) Phenotypes of wild-type and transgenic plants before drought treatment (top panel), after withholding water for 1 week (middle panel), and after recovery for 3 d (bottom panel). (B) Survival of transgenic and wild-type plants. Data are means ±SD from three independent biological replicates. Asterisks indicate statistically significant differences compared with the control (Student’s *t*-test: **P<0.01). (C) Water loss rate from transgenic and wild-type plants. Water loss was expressed as a percentage relative to the total water content. Error bars are based on three replicates. For each repeat, 20 fully expanded leaves from ~4-week-old plants were used.

Because the expression level of *LP2* was inhibited by PEG and ABA treatment, the effect of *LP2* overexpression on plant drought sensitivity was next tested. The full-length open reading frame of *LP2* under control of the maize ubiquitin promoter was transformed into japonica rice cultivar Nipponbare. Fifteen independent transgenic lines were identified and hygromycin-resistant T_2_ plants were then screened and confirmed by qRT–PCR analysis (Supplementary Fig. S1 at *JXB* online). Three independent transgenic lines (OE-2, OE-8, and OE-24) with a high level of expression of *LP2* were chosen for further investigation. Four-week-old plants were subjected to drought stress (Fig. 4A, upper panel). After 8 d of drought treatment, the leaves of the control showed only slight rolling and wilting, but leaves of the three transgenic lines exhibited severe drought-induced rolling and wilting (Fig. 4A, middle panel). After re-watering for 2 d, growth of control plants was almost identical to that of the non-stressed control, whereas growth of the transgenic lines remained severely inhibited (Fig. 4A, bottom panel). Furthermore, the survival rates of OE-2, OE-8, and OE-24 plants were markedly lower than those of the controls. After rehydration, survival rates were only 13, 35, and 20% in OE-2, OE-8, and OE-24 lines, respectively, compared with 85% for the control (Fig. 4B). The results indicated that overexpression of *LP2* reduced drought tolerance in rice. The rate of water loss from detached leaves of the wild-type and *LP2* overexpression lines was then measured. The overexpression lines lost water much faster than wild-type plants (Fig. 4C). Thus LP2 was shown to be a negative regulator of the drought signalling pathway.

### *LP2* overexpression results in decreased stomatal closure and increased stomatal density

**Fig. 5.**
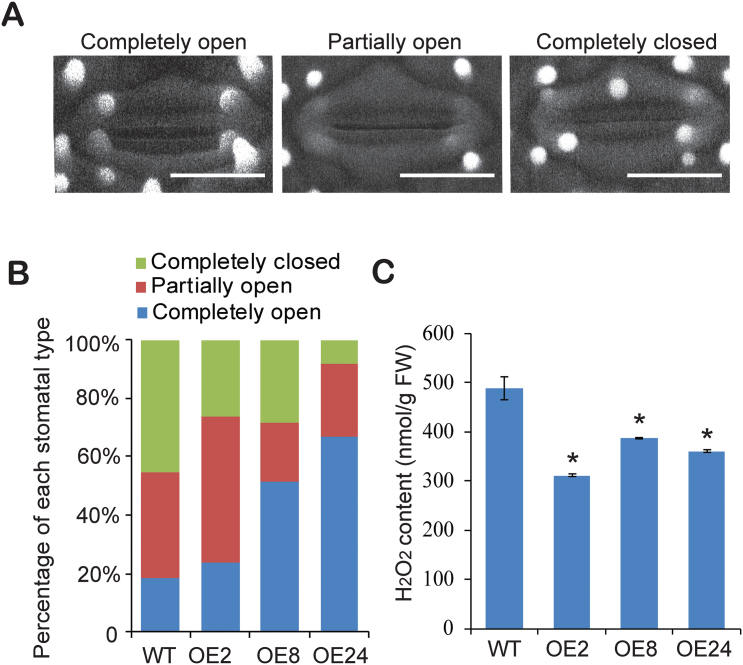
Down-regulation of H_2_O_2_ in *LP2*-overexpressing plants inhibits stomatal closure. (A) Scanning electron microscopy images of three levels of stomatal opening. Scale bar=10 μm. (B) Percentages of three levels of stomatal opening in wild-type and *LP2*-overexpressing plants (*n*=63 stomata for the wild type; *n*=55 stomata for OE-2; *n*=72 stomata for OE-8; *n*=42 stomata for OE-24). (C) Quantitative measurements of H_2_O_2_ in seedling leaves of wild-type and *LP2*-overexpressing plants. Data are means ±SD from three independent biological replicates. Asterisks indicate statistically significant differences compared with the control (Student’s *t*-test: *P<0.05).

Plant response to drought stress is closely associated with stomatal movement (Hetherington and Woodward, 2003). It was therefore investigated whether the altered phenotypes of *LP2* overexpression plants under drought stress were correlated with differences in stomatal aperture by environmental scanning electron microscopy; 45.5% of stomata were completely closed in wild-type plants under drought stress, whereas only 26, 28.6, and 8.3% were completely closed in *LP2*-overexpressing lines OE-2, OE-8, and OE-24, respectively (Fig. 5A, B). In addition, 36.4% of stomata were partially open in the wild-type plants, but 50.0, 20.0, and 25.0% of stomata were partially open in the *LP2*-overexpressing lines OE-2, OE-8, and OE-24, respectively (Fig. 5B). Furthermore, 18.2% of stomata were completely open in the wild-type plants, compared with 24.3, 51.4, and 66.7% in the respective *LP2*-overexpressing lines (Fig. 5B). These results suggested that increased drought sensitivity in *LP2*-overexpressing plants was largely due to enhanced stomatal opening. Since H_2_O_2_ induces stomatal closure (McAinsh *et al.*, 1996; Hamilton *et al.*, 2000; Apel and Hirt, 2004; Mittler *et al.*, 2004; Bright *et al.*, 2006), H_2_O_2_ contents in leaves of *LP2* overexpression lines were determined and lower accumulation relative to the control was found (Fig. 5C). These results suggested that the increased stomatal opening in *LP2* overexpression plants under drought stress may be due to decreased reactive oxygen (ROS) production.

### *LP2* expression is directly regulated by DST

**Fig. 6.**
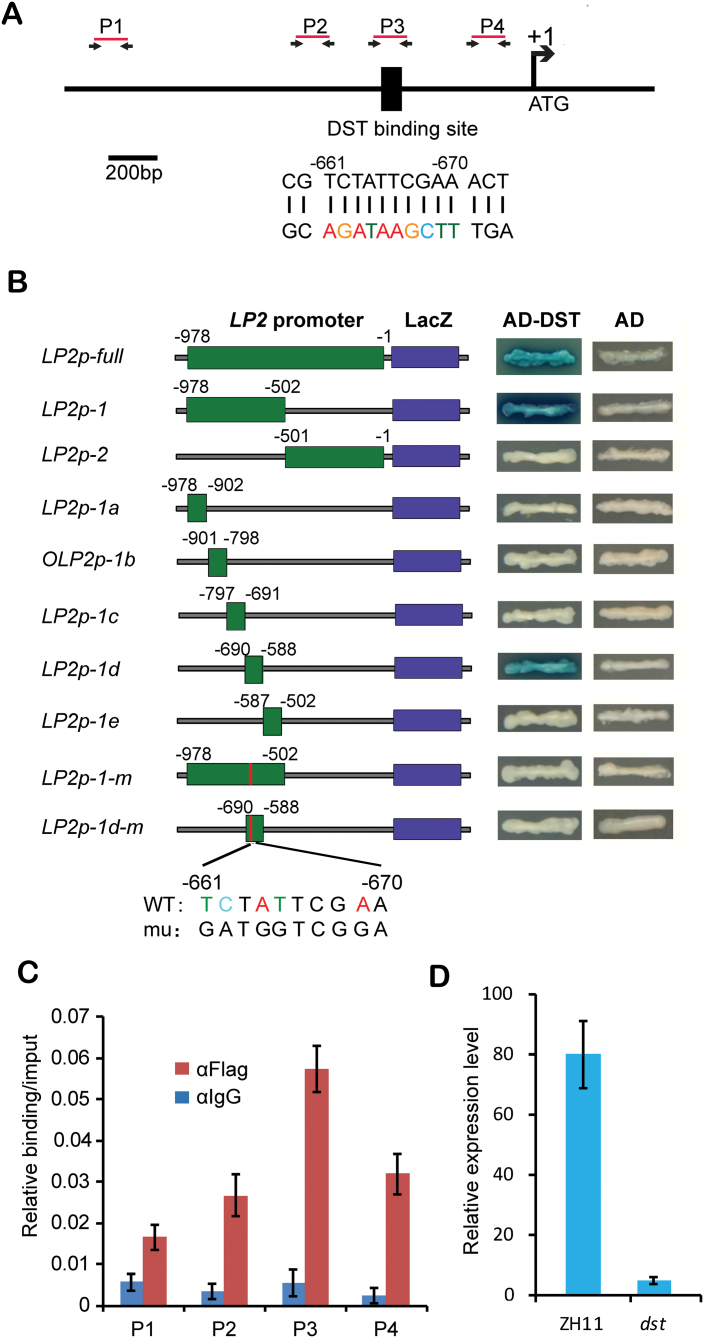
*LP2* is transcriptionally regulated by DST via direct binding to the promoter. (A) Schematic diagram of the promoter regions of *LP2*. A black line represents the promoter region of *LP2*; the black box on the line represents the putative DST-binding site. Upper numbers indicate relative distances from the ATG initiation codon shown as +1 (scale bars=200bp). (B) Yeast one-hybrid assays showing that DST activates the LacZ reporter gene driven by the *LP2* promoter containing the putative DST-binding motif, but not LacZ reporter genes driven by the *LP2* promoter without the binding motif, or with mutations in the binding motif. Red bars in the construct of LP2p-1-m and LP2p-1d-m indicate the positions of mutations. AD, activation domain. (C) ChIP-qPCR assays showing that *LP2* promoter fragments containing the putative DST-binding site are specifically enriched. Four pairs of primers were used for the ChIP-qPCR experiment (means ±SD, *n*=3). Immunoprecipitation with a pre-immune (Pre.) serum was used as the negative control. (D) Comparison of transcript abundance of *LP2* in the wild type (ZH11) and *dst* mutant by qRT–PCR.

Analysis of the promoter sequence of *LP2* revealed a DST-binding element (DBE) at position –670 to –661 (Huang *et al.*, 2009; Fig. 6A). DST is a zinc finger transcription factor that negatively regulates stomatal closure by direct modulation of genes related to H_2_O_2_ homeostasis (Huang *et al.*, 2009). To test the possibility that DST directly binds to the DBE in the *LP2* promoter, a yeast one-hybrid assay was performed. The results showed that DST protein binds directly to the promoter sequence of *LP2* (–2126 to –1) (Fig. 6B). To determine whether the predicted DBE is functional, several truncation derivatives of the –2126 to –1 promoter sequence were subjected to transactivity assays. As shown in Fig. 6B, only the fragment including the DBE interacted with DST in yeast. When the DBE was deleted or mutated, there was no binding (Fig. 6B). A chromatin immunoprecipitation-quantitative real-time PCR (ChIP-qPCR) assay was further applied to investigate the interaction between *DST* and the *LP2* promoter. The results indicated a significant enrichment of DST binding to the DBE of the *LP2* promoter, in contrast to its negligible binding to other positions (Fig. 6C). The expression level of *LP2* in the *dst* mutant was also assayed and, as shown in Fig. 6D, expression of *LP2* was greatly decreased in the mutant. These results indicated that DST bound to the DBE at position –670 to –661 of the *LP2* promoter to regulate *LP2* expression directly.

### LP2 physically interacts with plasma membrane aquaporins

**Fig. 7.**
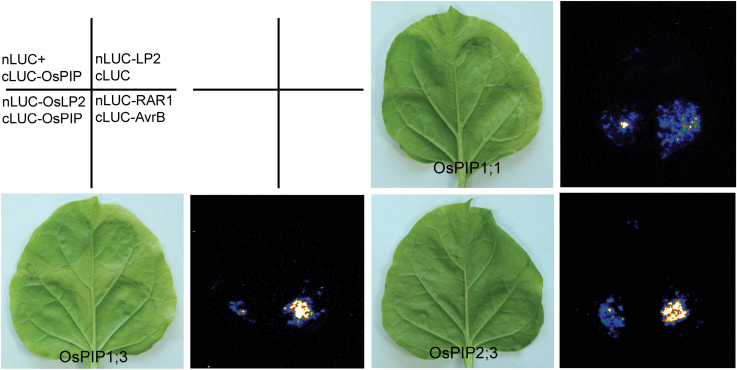
LP2 physically interacts with plasma membrane aquaporins. Interaction of LP2 with three aquaporins as indicated by firefly luciferase complementation imaging assays in *Nicotianana benthamiana* leaves. nLUC-RAR1 and cLUC-AvrB were used as positive controls. nLUC and cLUC-OsPIP as well as nLUC-LP2 and cLUC were used as negative controls.

In rice, several aquaporins including OsPIP1; 1, OsPIP1; 3, and OsPIP2; 3 were shown to play crucial roles in response to drought stress by transgenic analyses (Lian *et al.*, 2004; Yu *et al.*, 2006). Recently, SIRK1, a member of the LRR-RLK family in *Arabidopsis*, was shown to interact with, and activate, multiple aquaporins via phosphorylation (Wu *et al.*, 2013). To investigate if *LP2* interacted with these aquaporins, Nluc-LP2 and Cluc-PIP vectors were constructed for firefly luciferase complementation imaging assays (Chen *et al.*, 2008). Co-expression of NLuc-LP2 and cLUC-OsPIP1; 1 resulted in strong LUC complementation, similar to NLuc-LP2/cLUC-OsPIP1; 3, NLuc-LP2/cLUC-OsPIP2; 3, and the positive control, whereas co-expression of NLuc/cLUC-OsPIP or NLuc-LP2/Cluc led to negligible LUC activity (Fig. 7). To evaluate if LP2 can specifically phosphorylate theses aquaporins *in vitro*, attempts were made to express OsPIP1; 1, OsPIP1; 3, and OsPIP2; 3 in *E. coli*; these failed however. New measures need to be taken to address this problem.

## Discussion

Unlike animals, higher plants, which are sessile and cannot escape their surroundings, must adapt to environmental change by various molecular responses. For example, when water deficit occurs in the soil, plants respond by activating drought resistance pathways that induce or change the expression level of drought-responsive genes that produce transcription factors, protein kinases, and osmoprotectant-synthesizing enzymes (Hadiarto and Tran, 2011). RLKs are signalling proteins that perceive environmental signals by extracellular domains and transduce them into the cell via a transmembrane domain and an intracellular kinase domain (Morillo and Tax, 2006). RLKs convey signals to target proteins in the cytoplasm by catalytic processes of protein kinase activity. In plants, RLKs comprise a large gene family and regulate various plant processes, including growth and development as well as abiotic stresses (Osakabe *et al.*, 2013). *RLK7* (*RECEPTOR-LIKE KINASE 7*) encoding an LRR-RLK is implicated in control of germination speed and tolerance to oxidative stress (Pitorre *et al.*, 2010). *Arabidopsis RPK1* (*RECEPTOR-LIKE KINASE 1*) participates in early ABA signalling as well as abiotic stress responses; overproduction of *RPK1* leads to enhanced drought tolerance accompanied by enhanced expression of stress- and H_2_O_2_-responsive genes (Osakabe et al., 2005, 2010). *OsSIK1* and *OsSIK2* overexpression results in enhanced drought tolerance in rice (Ouyang *et al.*, 2010; Chen *et al.*, 2013). Although the role of the RLK genes in regulating the environmental stress response is well recognized in plants, less is known about the regulatory mechanism of these genes. In the present study, it was shown that the LRR-RLK gene *LP2* was transcriptionally regulated by the C_2_H_2_ zinc finger transcription factor DST, via direct binding to its promoter. Expression of *LP2* was reduced after salt, ABA, and PEG treatments. Overexpression of *LP2* in rice reduced the H_2_O_2_ content and stomatal closure, resulting in drought sensitivity of transgenic plants. Furthermore, it was found that LP2 interacted *in vivo* with OsPIPs. These findings established a new branch in the pathway of DST-mediated drought sensing pathway in plants.

Expression of *LP2* was previously found to be extremely down-regulated in the *dst* mutant (Huang *et al.*, 2009). DST is a zinc finger transcription factor that regulates drought and salt tolerance by controlling H_2_O_2_-induced stomatal closure. This suggested the possibility that LP2 played a role in abiotic stress response and acted downstream of DST. In the present study, analysis of the *LP2* promoter revealed a DBE in the upstream region of –670 to –661, and a yeast one-hybrid assay showed that only the fragment including the DBE can interact with DST in yeast, suggesting that *LP2* transcription is likely to be directly regulated by DST. In addition, *LP2* was negatively regulated by drought stress, consistent with the induction expression pattern of *DST* (Fig. 2A; Huang *et al.*, 2009). Overproduction of *LP2* in transgenic plants led to drought sensitivity and a faster rate of water loss, corresponding to lower stomatal closure and H_2_O_2_ content (Figs 4, 5). H_2_O_2_ is as an important signalling molecule that plays a key role in induction of leaf stomatal closure (McAinsh *et al.*, 1996; Hamilton *et al.*, 2000; Apel and Hirt, 2004; Mittler *et al.*, 2004; Bright *et al.*, 2006; Tan *et al.*, 2014). Thus, LP2 functions as a negative regulator of drought tolerance. The present studies added a new member to the DST-mediated drought signalling pathway.

So far, only two LRR-RLK family genes, *OsSIK1* and *OsSIK2*, affecting drought response have been reported in rice (Ouyang *et al.*, 2010; Chen *et al.*, 2013). This study reports a third gene, *LP2*, also involved in drought response. This finding adds further support to the central role of LRR-RLK proteins in regulating drought response in a staple crop plant. However, there are several important differences in regard to *LP2* compared with *OsSIK1* and *OsSIK2*. First, although the three proteins all belong to the LRR-RLK gene family, LP2 does not share significant primary sequence homology with OsSIK1 and OsSIK2 (amino acid identities are only 30.27% and 18.04%, respectively). Phylogenetic analysis revealed that the three proteins are in different subgroups (Fig. 1B). Secondly, *LP2* is mainly expressed in leaves and other green tissues, and its expression is reduced by drought, ABA, and PEG treatments (Fig. 2), whereas *OsSIK1* is mainly expressed in the stem and spikelet, and *OsSIK2* is expressed in the leaf and leaf sheath, and their expression levels are induced by NaCl and drought treatments. Thirdly, although transgenic plants overexpressing each gene accumulate reduced levels of H_2_O_2_ in leaves, they show different phenotypes; *LP2*-overexpressing plants are sensitive to drought stress (Fig. 4), whereas *OsSIK1*- and *OsSIK2*-overexpressing plants show higher tolerance to drought stress than control plants. These results indicate that LP2 and the other two OsSIK genes may play different roles in drought response.

Discovery of aquaporins in plants led to a paradigm shift in the understanding of water relations. A large number of plant aquaporins were identified and explained according to their importance in regulating water flow through membranes and in maintaining cellular homeostasis at all developmental stages and under different environmental conditions (Hachez *et al.*, 2006; Chaumont and Tyerman, 2014). However, water is not the sole molecule that diffuses through aquaporins. Accumulating evidence indicates that aquaporins represent an important membrane-selective pathway for diffusion of several neutral solutes, including glycerol, urea, ammonia, carbon dioxide, H_2_O_2_, and the metalloids boric acid, silicic acid, and arsenite (Tyerman *et al.*, 2002; Maurel *et al.*, 2008; Gomes *et al.*, 2009; Hachez and Chaumont, 2010; Ma, 2010; Miwa and Fujiwara, 2010; Bienert and Chaumont, 2014; Chaumont and Tyerman, 2014; Kaldenhoff *et al.*, 2014). These solutes are essential for plant growth and development, thus suggesting a role for aquaporins in these processes. Transgenic analyses showed that several aquaporins also have confirmed roles in response to abiotic stress, including drought, salt, and low temperature stresses (Siefritz *et al.*, 2002; Lian *et al.*, 2004; Yu *et al.*, 2006; Prak *et al.*, 2008; Matsumoto *et al.*, 2009; Liu *et al.*, 2013; Li *et al.*, 2014). PIPs represent one subfamily of aquaporins and comprise 11 members in rice. Transgenic analyses revealed that multiple rice PIPs, including OsPIP1; 1, OsPIP1; 3, and OsPIP2; 3, play important roles in drought response (Lian *et al.*, 2004; Yu *et al.*, 2006). In addition, PIP activities are regulated by post-translational modifications and protein interactions (Chaumont and Tyerman, 2014). By using a LUC assay in the present study, it was shown that LP2 interacted with these three PIPs in tobacco cells (Fig. 7). This indicated that these aquaporins might be phosphorylation targets of LP2. More recently, SIRK1, a member of the LRR-RLK family in *Arabidopsis*, was identified to undergo rapid transient phosphorylation after resupply of sucrose to sucrose-starved seedlings, and shown to interact directly with aquaporins to activate them via phosphorylation (Wu *et al.*, 2013). It was believed that LP2 regulated the activity of OsPIP1; 1, OsPIP1; 3, and OsPIP2; 3 in a similar way, but the hypothesis could not be confirmed in an *in vitro* kinase assay, because of failure in expressing the three PIP proteins. The biological meaning of LP2–OsPIP interactions remains to be further investigated.

It is well known that the phytohormone ABA is an important regulator of plant response to abiotic stress such as drought by mediating stomatal closure. The main molecular mechanism was proposed as follows: the ABA produced binds to ABA PYR (pyrabactin resistance) receptors and inhibits the activity of PP2C protein phosphatases, including ABI1, which acts as the suppressor of downstream key protein kinases, such as SnRK2 kinases; in turn, ABA activates the S-type anion channel SLAC1 via SnRK2 kinases (Schmidt *et al.*, 1995; Park *et al.*, 2009; Hubbard *et al.*, 2010). However, some stress response pathways are independent of ABA (Zhu, 2002; Yamaguchi-Shinozaki and Shinozaki, 2006). It was reported that the DST-mediated signalling pathway was ABA independent (Huang *et al.*, 2009). Thus, in the present study LP2, through direct transcriptional regulation by DST, participated in drought stress signalling most probably in an ABA-independent way. In addition, unlike the above ABA–PYR–PP2C–SnRK2–SLAC1 pathway, which promotes stomatal closure, the DST–LP2 pathway enhances stomatal opening by reduction of H_2_O_2_ accumulation. Therefore, under normal conditions, it is possible that DST promotes transcription of LP2, leading to the stomatal opening and in turn promoting plant growth. When plants are exposed to abiotic stresses, the active ABA content level is elevated and positive regulatory pathways are activated, resulting in stomatal closure. At the same time, expression of LP2 is significantly inhibited by stress conditions (Fig. 2A) in a similar manner to its upstream regulator DST (Huang *et al.*, 2009). Therefore, the two pathways may act co-ordinately to maintain plant growth and development.

## Supplementary data

Supplementary data are available at *JXB* online.

Figure S1. Expression levels of the *LP2* gene in WT and transgenic lines.

Table S1. Primers used in this study.

Supplementary Data
