# Supplementary material for: Plasma membrane receptor-like kinase leaf panicle 2 acts downstream of the DROUGHT AND SALT TOLERANCE transcription factor to regulate drought sensitivity in rice
Source: J Exp Bot. 2014 Nov 10;66(1):271–81. doi: 10.1093/jxb/eru417 (PMC4265162; doi:10.1093/jxb/eru417)
Supplement: Supplementary Data [file supp_66_1_271__index.html]

Plasma membrane receptor-like kinase leaf panicle 2 acts downstream of the DROUGHT AND SALT TOLERANCE transcription factor to regulate drought sensitivity in rice — Plasma membrane receptor-like kinase leaf panicle 2 acts downstream of the DROUGHT AND SALT TOLERANCE transcription factor to regulate drought sensitivity in rice — Supplementary Data 

# Plasma membrane receptor-like kinase leaf panicle 2 acts downstream of the DROUGHT AND SALT TOLERANCE transcription factor to regulate drought sensitivity in rice

## Supplementary Data

Data files

**Files in this Data Supplement:**

- Supplementary Data - Supplementary Data
