# Supplementary material for: Plasma membrane receptor-like kinase leaf panicle 2 acts downstream of the DROUGHT AND SALT TOLERANCE transcription factor to regulate drought sensitivity in rice
Source: J Exp Bot. 2014 Nov 10;66(1):271–81. doi: 10.1093/jxb/eru417 (PMC4265162; doi:10.1093/jxb/eru417)
Supplement: Supplementary Data [file supp_eru417_jexbot134163_file001.pdf]

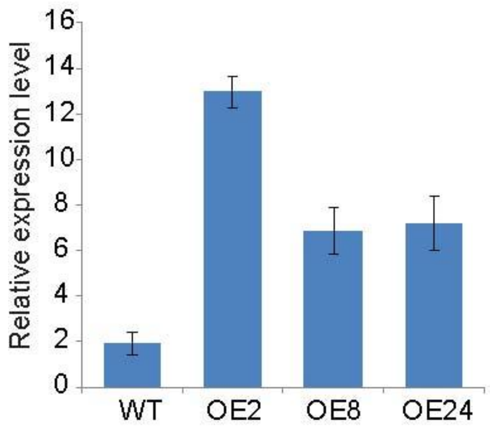

Figure S1 Expression levels of the *LP2* gene in WT and transgenic lines.

Table S1. Primers used in this study

Prime name	Prime sequence (5'-3')	Description
LP2-over- F	TCTGCACTAGGTACCTGCAG ATGGGCCTCACGTGTGATACAC	LP2 overexpression
LP2-over- R	ATGGATCCGTCGACCTGCAG TGCATCTTGTTCATTACATGCC	
DST-ov-F	TCTGCACTAGGTACCTGCAG ATGGACTCCCCGTCGCCTATGG	DST overexpression
DST-ov-R	ATGGATCCGTCGACCTGCAG GAGGCTCAAGTTGAGGTCGA	
LP2-580-F	CGGTCCCGGGGGATCCATGGGCCTCACGTG TGATA	LP2 subcellular localization
LP2-580-R	TGCTCACCATGGATCCTGCATCTTGTTCATT ACATGCC	
LRR-k-F:	TCGCGGATCCGAATTCGGTGAGAATTTCCT TAAAGTC	Protein expression in E.Coli
LRR-k-R:	GACGGAGCTCGAATTCTCAACCATATTTCTT AGCTTCCAATC	
CHIP-F1	AGGTGATGTTTCGAATCTATACCAA	CHIP-qPCR
CHIP-R1	GTGCCATGACGACGATGTGT	
CHIP-F2	TGTGATGAACTACGCCAAGACTATG	
CHIP-R2	CCCAATGAACAGGTCGAAAGC	
CHIP-F3	GAACGAGAATCTTGAAAGGTAAACC	
CHIP-R3	CCGGAACCGGACAGAGAAAT	
CHIP-F4	CCGAAGGTGCTCCATTCTCT	
CHIP-R4	AATATTCGCACTTCACTTTGAGC	
PB42AD-DST-F	TGCCTCTCCCGAATTCATGGACTCCCCGTC GCCTATGG	Yeast one-hybrid assays
PB42AD-DST-R	CGAGTCGGCCGAATTCCTAGAGGCTCAAGT TGAGGTCGA	
PLaczi –LRR-F	ATCTGTCGACCTCGAGCTTGGTCTAATTTT TTTTTTGCTAC	
PLaczi –LRR-R	GAGCACATGCCTCGAGGTTACCTGCAAAA ATATACAACC	
qLP2-F	ATGGGCCTCACGTGTGATAC	LP2 qPCR
qLP2-R	CAAGAAGGGCATCACCAACG	
